# Assessing Noncoding Sequence Variants of *GJB2* for Hearing Loss Association

**DOI:** 10.4061/2011/827469

**Published:** 2011-10-05

**Authors:** T. D. Matos, H. Simões-Teixeira, H. Caria, R. Cascão, H. Rosa, A. O'Neill, Ó. Dias, M. E. Andrea, D. P. Kelsell, G. Fialho

**Affiliations:** ^1^Centre for Biodiversity, Functional, and Integrative Genomics (BioFIG), Faculty of Science, University of Lisbon, Campus FCUL, Campo Grande, 1749-016 Lisboa, Portugal; ^2^Higher School of Health, Polytechnic Institute of Setúbal, Campus IPS, Estefanilha, 2914-503 Setúbal, Portugal; ^3^ORL Service, Hospital Garcia de Orta, EPE, Av. Torrado Silva, 2805-267 Almada, Portugal; ^4^Department of Head and Neck, Hospital Egas Moniz, CHLO, EPE, R. da Junqueira 126, 1349-019 Lisboa, Portugal; ^5^ORL Service, Hospital Santa Maria, CHLN, EPE, Av. Professor Egas Moniz, 1600 Lisboa, Portugal; ^6^Centre for Cutaneous Research, Blizard Institute, Barts and the London School of Medicine and Dentistry, University of London, London E1 2AT, UK

## Abstract

Involvement of *GJB2* noncoding regions in hearing loss (HL) has not been extensively investigated. However, three noncoding mutations, c.-259C>T, c.-23G>T, and c.-23+1G>A, were reported. Also, c.-684_-675del, of uncertain pathogenicity, was found upstream of the basal promoter. We performed a detailed analysis of *GJB2* noncoding regions in Portuguese HL patients (previously screened for *GJB2* coding mutations and the common *GJB6* deletions) and in control subjects, by sequencing the basal promoter and flanking upstream region, exon 1, and 3'UTR. All individuals were genotyped for c.-684_-675del and 14 SNPs. Novel variants (c.-731C>T, c.-26G>T, c.*45G>A, and c.*985A>T) were found in controls. A hearing individual homozygous for c.-684_-675del was for the first time identified, supporting the nonpathogenicity of this deletion. Our data indicate linkage disequilibrium (LD) between SNPs rs55704559 (c.*168A>G) and rs5030700 (c.*931C>T) and suggest the association of c.[*168G;*931T] allele with HL. The c.*168A>G change, predicted to alter mRNA folding, might be involved in HL.

## 1. Introduction

About two hundred *GJB2* mutations causing nonsyndromic hearing loss (NSHL) have been reported (http://www.hgmd.org/) [1]. Most* GJB2* mutations described so far localize to the coding region (totally included within exon 2), which is routinely analysed upon the study of *GJB2* in HL patients. Also involved in HL, two deletions, del(*GJB6*-D13S1830) [2–4] and del(*GJB6*-D13S1854) [5] disrupt the *GJB6* gene which codes for connexin-30, and it is thought that they may ablate a *GJB2 cis*-regulatory sequence [5–7]. This putative element is likely to be ablated by a third deletion, del(chr13 : 19,837,344–19,968,698), localized upstream of *GJB2* and *GJB6* [8, 9].

Along with *GJB2* coding region, the noncoding first exon and donor splice site have been analysed in several studies, and two pathogenic mutations, c.-23G>T (exon 1) [10] and c.-23+1G>A (intron) [11], both in the donor splice site, have been identified. The c.-23+1G>A mutation (commonly known as IVS1+1G>A), shown to impair splicing [12], has been identified in several cases, being particularly frequent in Czech Republic, Turkey, and Hungary [13–15].

A few studies have investigated, in addition to exon 1, the noncoding region immediately upstream of this exon, including the basal promoter [14, 16–21].

Houseman and coworkers [16] analysed HL patients heterozygous for c.101T>C (p.Met34Thr), in which no second *GJB2* coding mutation had been detected, and identified a monoallelic 10 bp deletion, c.-684_-675del (firstly designated -493del10), upstream of the basal promoter. The deletion was also present in other hearing impaired individuals as well as in control individuals, with or without c.101T>C. However, c.-684_-675del homozygosity was only observed in c.101T>C homozygous patients. The fact that in the control population 22 of the 25 (88%) c.101T>C heterozygotes carried the deletion suggested the existence of LD between c.101T>C and c.-684_-675del, later demonstrated by Zoll and coworkers [22]. Transcription was observed from alleles harbouring in *cis* the deletion and the variant c.101T>C, derived from keratinocytes and cell lines. However, eventual subtle differences would not have been detected, since this was not a quantitative analysis [16]. To date, the role of c.-684_-675del in HL has remained uncertain.

More recently, a pathogenic basal promoter mutation, c.-259C>T (firstly designated -3438C>T) was identified, in *trans* with c.250G>A (p.Val84Met), in a Portuguese HL patient, highlighting the relevance of screening *GJB2* noncoding regions in nonelucidated cases [18].

In the present study, we have analysed the basal promoter and the flanking upstream region, as well as the exon 1 and the 3′UTR of the *GJB2 *gene in 89 Portuguese HL patients. The same analysis was conducted on 91 normal hearing control individuals from the Portuguese population.

## 2. Methods

### 2.1. Subjects

Eighty-nine Portuguese HL patients previously screened for mutations in the *GJB2 *coding region and acceptor splice site (by SSCP and/or sequencing) and for the del(*GJB6*-D13S1830) and del(*GJB6*-D13S1854) *GJB6* deletions (using the methodology described in [5]) were enrolled in this study. Eight patients were heterozygous for a *GJB2* coding mutation: c.71G>A (p.Trp24X; *n* = 1), c.35delG (*n* = 3), c.109G>A (p.Val37Ile; *n* = 1), c.380G>A (p.Arg127His; *n* = 1), c.457G>A (p.Val153Ile; *n* = 2), and one patient was heterozygous for the c.-22-12C>T variant (apparently a polymorphism; dbSNP accession number rs9578260). No patient harboured either of the known *GJB6* deletions. The HL was nonsyndromic in all patients, except for one of them, who presented with Waardenburg syndrome. The patient was heterozygous for the controversial c.457G>A mutation and was thus included in the study. The patients presented with bilateral, mild to profound HL, and were either familial or sporadic cases. The familial cases predominantly showed a recessive pattern of inheritance. All patients were audiologically evaluated by pure tone audiometry.

The control sample was composed of 91 Portuguese individuals with apparent normal hearing. The status regarding c.101T>C *GJB2* variant of those control individuals harbouring the c.-684_-675del, here referred, had been previously investigated, by sequencing, as part of an unpublished work. The status of the entire *GJB2* coding region is not known for the vast majority of the 91 control individuals, which were blindly included in this study (and not based on their eventually available *GJB2* coding region status).

Informed consent was obtained from all the participants.

### 2.2. Genetic Analysis

In all individuals, we have sequenced a region of about 0.7 kb immediately upstream of the exon 1 (which includes the basal promoter), the exon 1, and the whole 3′UTR. The region upstream of the exon 1, plus exon 1 and donor splice site, was amplified in a 1009 bp amplicon, using the pair of primers PF2 5′-CgTTCgTTCggATTggTgAg-3′ and PR1 5′-CAgAAACgCCCgCTCCAgAA-3′, as previously described [18]. The amplicons were sequenced using the primers PF2 and PF1 5′-ggCTCAAAggAACTAggAgATCg-3′. When necessary, primers PR1 and PR2 5′-ggAgACTgggAAAgTTACgg-3′ were used for sequencing. The 3′UTR (plus the last 90 nucleotides and stop codon) was amplified in 3 overlapping fragments using the following three pairs of primers: 3UTRaF 5′-gCAgTgTCTggAATTTgCATC-3′, 3UTRaR 5′-AggCACTggTAACTTTgTCC-3′, 3UTRbF 5′-CACgTTAAAggTgAACATTgg-3′, 3UTRbR 5′-CgACAgAAACTTCTCCCTC-3′, 3UTRcF 5′-gTAgCCAgCATCggAAAgAAC-3′, 3UTRcR 5′-ACTCTggCAACTTACCCATTg-3′. The 3′UTR PCR products were sequenced using the respective amplification forward primers.

### 2.3. DNA Sequence Variants and SNPs Description

Description of variants follows the HGVS recommendations, and is based on the *GJB2* reference sequences accessed through the following links:

1. https://research.cchmc.org/LOVD/refseq/GJB2_codingDNA.html;
2. https://research.cchmc.org/LOVD/refseq/GJB2_intron_01.html;
3. https://research.cchmc.org/LOVD/refseq/GJB2_upstream.html;
4. https://research.cchmc.org/LOVD/refseq/GJB2_downstream.html.

These sequences show 100% identity with the NM_004004.5 (link 1) and NG_008358.1 (links 2, 3, and 4) NCBI reference sequences.

Novel variants were submitted to dbSNP and the respective reference SNP (rs) accession numbers are provided within the text.

SNPs are referred to by the dbSNP reference SNP (rs) accession number whenever it was available, and by the HGVS recommended designation, relative to the forementioned reference sequences.

### 2.4. Genotyping and Statistical Analysis

We have genotyped all individuals for the c.-684_-675del deletion; three SNPs in the promoter (rs9550621 (c.-484T>C), rs73431557 (c.-410T>C), rs9552101 (c.-369A>G)); ten SNPs in the 3′UTR (c.*1C>T, rs3751385 (c.*84T>C), rs7337074 (c.*104A>T), rs7329857 (c.*111C>T), rs55704559 (c.*168A>G), rs5030700 (c.*931C>T), rs1050960 (c.*1067G>T), rs7623 (c.*1152G>A), rs11841182 (c.*1197T>A), and rs7988691 (c.*1277T>C)); one SNP downstream of the 3′UTR (rs11839674 (c.*1447G>A)).

For the sake of simplicity, when describing the composite genotypes regarding SNPs rs73431557 (c.-410T>C), rs3751385 (c.*84T>C), rs55704559 (c.*168A>G), and rs5030700 (c.*931C>T), the genotype at each position, indicated in order from 5′ to 3′, is designated by A, C, G, or T if homozygous, or by a code letter, according to IUPAC nucleotide ambiguity code, if heterozygous.

The allelic frequencies regarding deletion c.-684_-675del and the 14 SNPs, were determined in the control population and used to test for Hardy-Weinberg equilibrium. The chi-square test was used to compare the allelic frequencies of the patients with those of the normal hearing individuals. Allelic frequencies of the control sample for the 14 SNPs were used to calculate pairwise linkage disequilibrium values. Testing for Hardy-Weinberg equilibrium, calculation of pairwise linkage disequilibrium values, and haplotype estimation (through the expectation maximization algorithm), were performed using SNPAnalyzer 1.2A online software (http://snp.istech.info/snp/SNPAnalyzer.html).

### 2.5. Analysis of mRNA Folding

Mfold (http://mfold.rna.albany.edu/?q=mfold/RNA-Folding-Form) [23] was used to assess the effect of alleles c.[*168A;*931C], c.[*168G;*931T], c.[*168A;*931T], and c.[*168G;*931C] on the folding of *GJB2* mRNA (template sequence: ENST00000382848, retrieved from Ensembl). For each sequence the lowest free-energy structure was considered.

## 3. Results and Discussion

In the current study, 89 Portuguese HL patients, previously screened for mutations in the *GJB2 *coding region and acceptor splice site (80 patients presenting no mutation, plus eight heterozygous for coding mutations and one heterozygous for the noncoding variant c.-22-12C>T), and 91 hearing individuals were analyzed as regards the noncoding region immediately upstream of the exon 1 (which includes the basal promoter), the exon 1, and the whole 3′UTR of *GJB2* gene. All individuals were also genotyped for c.-684_-675del and 14 SNPs localized therein.

### 3.1. DNA Sequence Variants

No additional *GJB2* variant was found in any of the eight patients previously found to be heterozygous for a coding *GJB2* mutation or in the patient heterozygous for the c.-22-12C>T noncoding variant.

Among the remaining 80 patients, six of them presented noncoding variants, which had already been reported (Table 1).

One patient, presenting with profound HL was heterozygous for the donor splice site c.-23+1G>A mutation. The patient may just be a carrier, or other *GJB2* or *GJB6* mutation might remain undetected. One other patient, presenting with moderate to severe HL, harboured in heterozygosity the c.-216T>G variant, located within the basal promoter, between two GT boxes [24, 25]. This variant was previously identified in two HL patients, also in heterozygosity [26]. The c.-45C>A variant in exon 1 was found in heterozygosity in one individual with severe HL. This variant was referred, by Wilch and coworkers [8], as an SNP at position +94 in exon 1. These authors observed expression of the *GJB2* allele harbouring the variant but, since a quantitative comparison with wild-type allele was not performed, a possible contribution to HL cannot be excluded. Three affected individuals (two heterozygous and one homozygous) harboured the deletion c.-684_-675del. 

No novel putative pathogenic noncoding mutation has been found in the patients, which might be due to the low number of monoallelic individuals and the small sample size. It is also possible that, simply, such mutations are very rare in our population. 

Among controls, four novel noncoding variants were identified: c.-731C>T, c.-26G>T, c.*45G>A, and c.*985A>T (rs112400198, rs112875543, rs112399473, and rs111729919, resp.). Each of these variants was identified only once, in heterozygosity, and in different individuals (Table 1). The hearing individual harbouring the novel c.-731C>T variant was also heterozygous for the recessive c.670A>C (p.Lys224Gln) mutation (https://research.cchmc.org/LOVD/; phase unknown). One control individual harboured the c.-45C>A exon 1 variant in heterozygosity (Table 1). Interestingly, we found one control subject homozygous for c.-684_-675del (Table 1), which is, to our knowledge, the first case described to date of a normal hearing individual presenting this genotype. This individual did not harbour the c.101T>C mutation. Our finding, together with the previous report of transcription from alleles harbouring c.-684_-675del [16] suggests the nonpathogenicity of the deletion. In addition, six normal hearing heterozygotes for the deletion were also identified (Table 1), with one also heterozygous for c.101T>C.

It should be noted that the pathogenic basal promoter mutation c.-259C>T, identified for the first time in a Portuguese family [18], was not found among the 89 patients and 91 normal hearing individuals here analysed, and neither was it identified in the other studies which analysed the basal promoter [14, 16–21]. Therefore, known occurrence of c.-259C>T continues to be restricted to that Portuguese family.

### 3.2. Genotypic Data and Statistical Analysis

The allelic frequencies and Hardy-Weinberg equilibrium status regarding the deletion c.-684_-675del and the 14 noncoding SNPs were determined (Table 2; see Supplementary Table  1 in Supplementary Material available online at doi: 10.4061/2011/827469).

The allelic frequencies of the deletion c.-684_-675del in patients and controls are not statistically different (Table 2). The allelic frequency observed for this deletion in our control population is close to the one found among the British control population [16], and higher than the one determined in the German control population [22].

The allelic frequencies regarding SNPs c.-410T*>*C, c.*84T*>*C, c.*168A*>*G, and c.*931C*>*T, were statistically different between patient and control groups (Table 2). 

**Figure 1:**
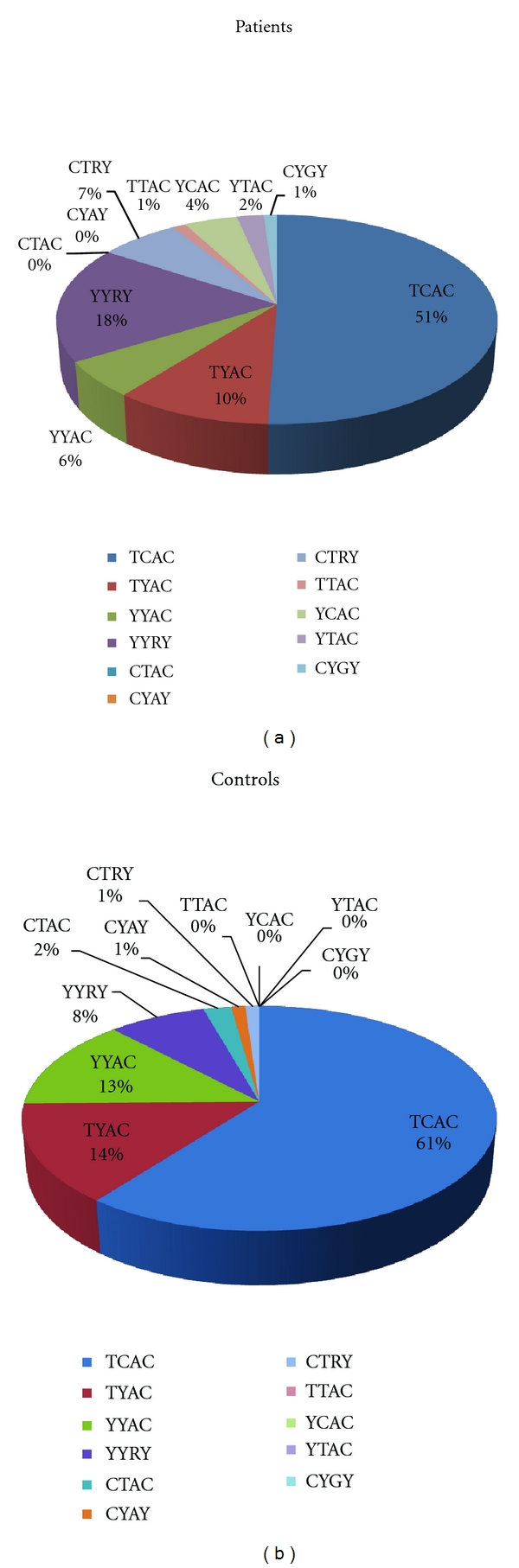
Frequencies, in patients (*n* = 89) and controls (*n* = 91), of the composite genotypes concerning SNPs c.-410T>C, c.*84T>C, c.*168A>G, and c.*931C>T; R = A/G heterozygosity; Y = C/T heterozygosity (based on IUPAC nucleotide ambiguity code).

By sorting both patients and controls into groups reflecting the genotypes for these four SNPs altogether, eleven composite genotypes were evidenced (Figure 1). Comparison of the genotypic frequencies in controls and patients promptly revealed an increased frequency in patients of the genotypes YYRY and CTRY, both heterozygous for SNPs c.*168A>G and c.*931C>T. Also, the genotype YCAC was identified in four patients but not found in controls. On the contrary, a decrease was observed in the frequency of the three genotypes that are most represented in controls—TCAC, TYAC, and YYAC. Each of the remaining genotypes was scarcely represented in both controls and patients (0%–2%), and their frequency did not vary more than 2% between the two groups; only 3% of controls and 4% of patients belong to one of these genotypes.

We also observed that, regarding SNPs c.*168A>G and c.*931C>T, nearly all individuals analysed (178/180) were either c.[=;=]+[=;=] or c.[*168A>G(+)*931C>T], which results from LD between these two SNPs (Supplementary Table  2, SNP pair 8 : 9). Interestingly, the overrepresentation of c.[*168A>G(+)*931C>T] genotype among patients, when comparing to hearing controls, is statistically very significant (*χ* ^2^ = 28.159; *P* = 3.4 E-06), thus accounting for the statistically significant differences in the allelic frequencies of these two SNPs between patients and hearing controls. 

The statistically significant differences also observed in the allelic frequencies of SNPs c.-410T>C and c.*84T>C seems to be due to the differential association of their variants with the estimated predominant alleles c.[*168A+*931C] and c.[*168G+*931T] (Tables 3(a) and 3(b)). This fact is in accordance with the observed LD between the two SNPs and SNPs c.*168A>G and c.*931C>T (Supplementary Table  2, SNP pairs 2 : 8, 2 :  9, 5 : 8, and 5 : 9).

It should be noticed that the presence of genotype YCAC among patients lends some contribute to the difference in allelic frequencies between patients and controls regarding SNP c.-410T>C.

The fact that YCAC genotype is not represented in 91 control individuals while it occurs in 4/89 patients is noteworthy. The presence of genotype YCAC implies the presence of haplotype CCAC, which frequency is of at least 2,2% among patients, and estimated to be null in the control population (as inferred from Table 3(a)). In order to validate a possible association of haplotype CCAC with HL analysis of larger samples of patients and normal hearing individuals is necessary. Interestingly, one of the four patients with the referred composite genotype is a c.457G>A heterozygote (phase unknown).

### 3.3. 3′UTR Variants and mRNA Folding

**Figure 2:**
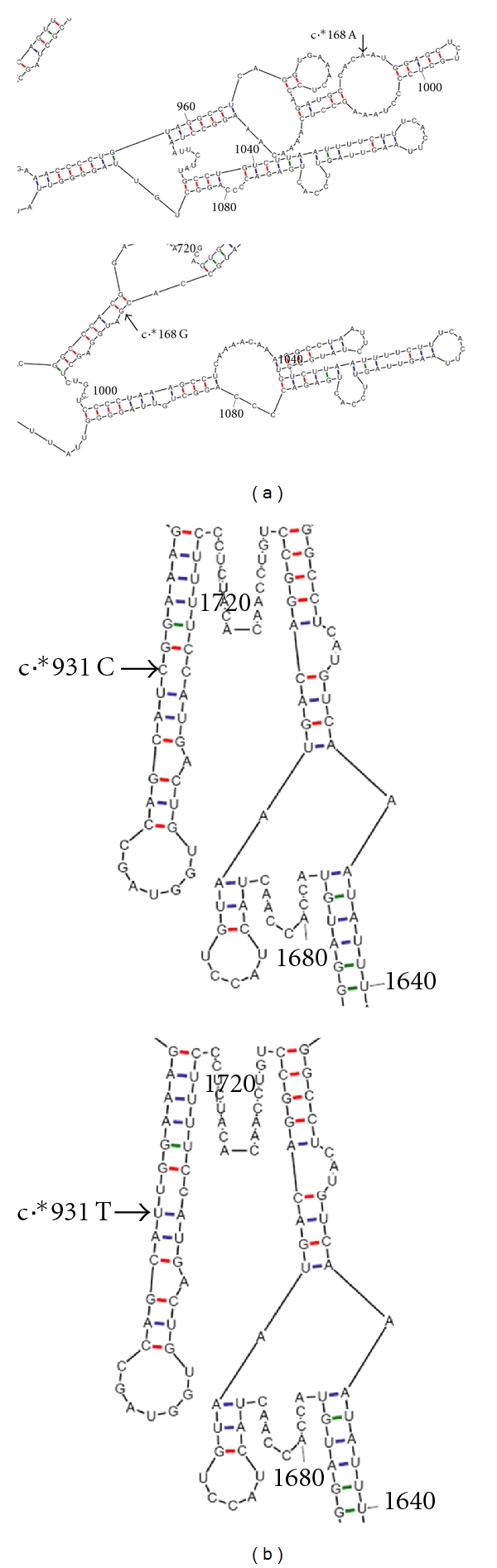
Effect of c.*168A>G and c.*931C>T changes in the 3′UTR on *GJB2* mRNA folding. (a) c.*168A and c.*168G; (b) c.*931C and c.*931T. The mRNA folding associated with each variant is the same regardless of the allele present at the other position, therefore only one example for each variant is provided.

Our findings suggest that the c.[*168G;*931T] allele might have a deleterious effect, contributing to HL. We have used Mfold [23] to predict the effect of alleles c.[*168A;*931C], c.[*168G;*931T], c.[*168A;*931T], and c.[*168G;*931C] on mRNA folding. The change c.*168A>G, regardless of genotype at position c.*931, was predicted to alter mRNA folding. On the contrary, the change c.*931C>T, regardless of genotype at position c.*168, is not predicted to alter mRNA folding (Figure 2). 

The c.*168A was predicted to be located in an internal loop of a stem-loop structure (Figure 2). Regulatory motifs in mRNA 3′UTR seem to function in the context of specific secondary structure [27]. Stem-loop structures occurring in the 3′UTR have been implicated in gene expression, with roles at the level of mRNA stability (e.g., the SLDE of G-CSF gene [28], the CDE of TNF-alpha gene [27, 29], the complex structure integrating three C-rich elements of alpha-globin gene, the histone mRNA 3′ terminal stem-loops, and the IRE of TFRC gene [27]) or translation (e.g., the common 30–37 nucleotide long element present in the target mRNAs of TIA-1, a translational repressor [30], and the SECIS element [27]). The disruption of the predicted stem-loop structure and/or other adjacent stem-loop structures (Figure 2), induced by the c.*168A>G change, might lead to deregulation of the *GJB2* gene expression, thus being a contributor to the hearing loss phenotype. It should be stressed that mRNA folding predictions are fallible. This fact notwithstanding, the simple change of sequence, without affecting the secondary structure, could conceivably disrupt a binding site for a trans-acting factor, also leading to gene expression deregulation. Regarding the c.*931C>T variant, despite the predictions that c.*931C occurs in a helix and that the change from C to T does not have structural implications, the in vivo situation might be different. Functional studies involving constructs containing a reporter gene's coding sequence fused with *GJB2* 3′UTR could help elucidating the functional significance of these two sequence variants.

In this study, of a total of 15 patients presenting either a *GJB2* coding mutation or a noncoding variant, 14 do not harbour either the c.*168A>G or the c.*931C>T changes, whereas one patient, heterozygous for the controversial c.380G>A mutation, is a compound heterozygote regarding SNPs c.*168A>G and c.*931C>T (phase unknown). Therefore, our data do not allow withdrawal of conclusions concerning a putative role of the two 3′UTR variants in the HL of some monoallelic patients. In this regard, the investigation of the genotypes regarding c.*168A>G and c.931C>T variants in larger samples of monoallelic patients would be interesting. Finally, the finding of one c.*168G homozygote (a c.*931C>T heterozygote, and carrying no *GJB2* sequence variant) in our patient cohort, might further support a possible role of c.*168G in HL.

## 4. Conclusion

This study suggests the association of the noncoding SNPs c.*168A>G and c.*931C>T with HL. The c.*168A>G change is predicted to alter mRNA folding, suggesting a putative role of this SNP in the pathology. Our data also point to a possible association with HL of the haplotype CCAC, comprising SNPs c.-410T>C, c.*84T>C, c.*168A>G, and c.*931C>T, respectively. However, this observation requires validation through analysis of a larger number of subjects. The technique of targeted sequence capture and massively parallel sequencing makes it very easy and cost-effective to screen large numbers of genes, and might cover noncoding sequences of some of them, such as *GJB2*. This approach could prove to be very useful for genetic diagnosis in cases of NSHL [31], with predictable benefits for genetic counselling of the affected families.

## Supplementary Material

Results of the analysis of Hardy-Weinberg equilibrium and pair-wise linkage disequilibrium regarding the c.-684_-675del and the 14 SNPs studied in this work.

## Figures and Tables

**Table 1:** *GJB2* variants identified in this study. Novel variants are in italic. BP: basal promoter; Ex 1: exon 1; DSS: donor splice site; Ex 2: exon 2; CR: coding region; 3′UTR: 3′ untranslated region.

Variant	Location	Patients (*n* = 89)		Controls (*n* = 91)	
		Heterozygote	Homozygote	Heterozygote	Homozygote
c.-731C>T	5′ of the BP	0	0	**1**	0
c.-684_-675del	5′ of the BP	**2**	**1**	**6**	**1**
c.-216T>G	BP	**1**	0	0	0
c.-45C>A	Ex 1	**1**	0	**1**	0
c.-26G>T	Ex 1	0	0	**1**	0
c.-23+1G>A	DSS	**1**	0	0	0
c.670A>C (p.Lys224Gln)	Ex 2 (CR)	0	0	**1**	0
c.*45G>A	3′UTR	0	0	**1**	0
c.*985A>T	3′UTR	0	0	**1**	0

**Table 2:** Differences in the allelic frequencies, regarding c.-684_-675del and 14 SNPs, between patient and control samples (chi-square test). ND: not determined: chi-square test could not be performed for SNPs with an expected value <5, or for SNPs which both alleles were observed in only one sample. Four SNPs present statistically significant differences in allelic frequencies between patients and controls (*P* values <0.05, in bold).

Variant/SNP	Alleles	Patients (*n* = 178 alleles)		Controls	*P* value
		Observed	Expected	(*n* = 182 alleles)	
c.-684_-675del	wt	174	170.18	174	0.162043
	c.-684_-675del	4	7.82	8	

rs9550621	c.-484 C	162	165.29	169	0.338941
	c.-484 T	16	12.71	13	

rs73431557	c.-410 C	41	26.41	27	**0.002089**
	c.-410 T	137	151.59	155	

rs9552101	c.-369 A	20	19.56	20	0.916103
	c.-369 G	158	158.44	162	

c.*1C>T	c.*1 C	177	178	182	ND
	c.*1 T	1	0	0	

rs3751385	c.*84 C	129	139.86	143	**0.04734**
	c.*84 T	49	38.14	39	

rs7337074	c.*104 A	174	177.02	181	ND
	c.*104 T	4	0.98	1	

rs7329857	c.*111 C	174	177.02	181	ND
	c.*111 T	4	0.98	1	

rs55704559	c.*168 A	154	170.18	174	**3.33E-09**
	c.*168 G	24	7.82	8	

rs5030700	c.*931 C	155	169.20	173	**9.18E-07**
	c.*931 T	23	8.80	9	

rs1050960	c.*1067 G	19	19.56	20	0.893155
	c.*1067 T	159	158.44	162	

rs7623	c.*1152 A	165	165.29	169	0.93373
	c.*1152 G	13	12.71	13	

rs11841182	c.*1197 A	2	0	0	ND
	c.*1197 T	176	178	182	

rs7988691	c.*1277 C	178	176.04	180	ND
	c.*1277 T	0	1.96	2	

rs11839674	c.*1447 A	2	0	0	ND
	c.*1447 G	176	178	182	

**Table:** (a)

Haplotype	Frequency
TCAC	0.7802
CTAC	0.0989
TTAC	0.0714
CTGT	0.0440
CCAT	0.0055

**Table:** (b)

Allele (c.*168; c.*931)	c.-410 T>C	c.*84T>C
AC	89.6% T	82.1% C
GT	100% C	100% T
