# Supplementary material for: Assessing Noncoding Sequence Variants of GJB2 for Hearing Loss Association
Source: Genet Res Int. 2011 Oct 5;2011:827469. doi: 10.4061/2011/827469 (PMC3335567; doi:10.4061/2011/827469)
Supplement: Supplementary file 1 — Results of the analysis of Hardy-Weinberg equilibrium and pair-wise linkage disequilibrium regarding the c.-684_-675del and the 14 SNPs studied in this work. [file 827469.f1.docx]

SUPPLEMENTARY TABLES

Supplementary table 1. Analysis of Hardy-Weinberg equilibrium for c.-684_-675del and 14 SNPs. Calculations were based on the control population. ND – Not determined due to lack of genetic variability regarding the concerned SNP.

| **Variant/SNP** | **P-value** |
| --- | --- |
| **c.-684_-675del** | 0.02972810 |
| **c.-484 T>C** | 0.39134875 |
| **c.-410 T>C** | 0.08837465 |
| **c.-369 A>G** | 0.32767174 |
| **c.*1C>T** | ND |
| **c.*84T>C** | 0.45797172 |
| **c.*104A>T** | 0.95828080 |
| **c.*111C>T** | 0.95828080 |
| **c.*168A>G** | 0.65802073 |
| **c.*931C>T** | 0.61631805 |
| **c.*1067G>T** | 0.91543627 |
| **c.*1152G>A** | 0.45797190 |
| **c.*1197T>A** | ND |
| **c.*1277T>C** | 0.91543627 |
| **c.*1447G>A** | ND |

Supplementary table 2. Analysis of pair-wise linkage disequilibrium regarding the 14 SNPs studied. Calculations were based on the control population. The p-value and the scores for five measures of linkage disequilibrium (D, D’, Delta, Delta squared and Fisher's) are presented for each pair of SNPs. Pair-wise linkage disequilibrium involving SNPs 4, 12 and 14 could not be determined due to lack of genetic variability regarding these SNPs. Correspondence: 1 - c.-484 T>C; 2 - c.-410 T>C; 3 - c.-369 A>G; 4 – c.*1C>T; 5 - c.*84T>C; 6 - c.*104A>T; 7 - c.*111C>T; 8 - c.*168A>G; 9 - c.*931C>T; 10 - c.*1067G>T; 11 - c.*1152G>A; 12 - c.*1197T>A; 13 - c.*1277T>C; 14 - c.*1447G>A.

| **SNPs Pair** | **P-value** | **D** | **D'** | **Delta (Δ)** | **Delta squared (Δ^2^)** | **Fisher's** |
| --- | --- | --- | --- | --- | --- | --- |
| **1:2** | 0.1099 | -0.0106 | -1.0000 | -0.1158 | 0.0134 | 1.1146 |
| **1:3** | 0.0000 | 0.0636 | 1.0000 | 0.7894 | 0.6231 | 2.0000 |
| **1:5** | 0.0000 | 0.0495 | 0.8812 | 0.4680 | 0.2190 | 2.0000 |
| **1:6** | 0.0000 | 0.0051 | 1.0000 | 0.2680 | 0.0718 | 2.0000 |
| **1:7** | 0.0000 | 0.0051 | 1.0000 | 0.2680 | 0.0718 | 2.0000 |
| **1:8** | 0.4167 | -0.0031 | -1.0000 | -0.0595 | 0.0035 | 1.5460 |
| **1:9** | 0.3875 | -0.0035 | -1.0000 | -0.0633 | 0.0040 | 1.5053 |
| **1:10** | 0.0000 | 0.0578 | 0.9094 | 0.7179 | 0.5153 | 2.0000 |
| **1:11** | 0.0000 | 0.0608 | 0.9167 | 0.9167 | 0.8403 | 2.0000 |
| **1:13** | 0.7716 | -0.0004 | -1.0000 | -0.0207 | 0.0004 | 1.8619 |
| **2:3** | 0.0390 | -0.0163 | -1.0000 | -0.1466 | 0.0215 | 1.0331 |
| **2:5** | 0.0000 | 0.1107 | 0.9495 | 0.7588 | 0.5758 | 2.0000 |
| **2:6** | 0.6723 | -0.0008 | -1.0000 | -0.0310 | 0.0010 | 1.8516 |
| **2:7** | 0.6723 | -0.0008 | -1.0000 | -0.0310 | 0.0010 | 1.8516 |
| **2:8** | 0.0000 | 0.0374 | 1.0000 | 0.5138 | 0.2639 | 2.000 |
| **2:9** | 0.0000 | 0.0421 | 1.0000 | 0.5465 | 0.2987 | 2.0000 |
| **2:10** | 0.0715 | 0.0144 | 0.1540 | 0.1297 | 0.0168 | 1.9467 |
| **2:11** | 0.1099 | -0.0106 | -1.0000 | -0.1158 | 0.0134 | 1.1146 |
| **2:13** | 0.5484 | -0.0016 | -1.0000 | -0.0440 | 0.0019 | 1.7246 |
| **3:5** | 0.0000 | 0.0470 | 0.5440 | 0.3660 | 0.1340 | 2.0000 |
| **3:6** | 0.0000 | 0.0049 | 1.0000 | 0.2115 | 0.0448 | 2.0000 |
| **3:7** | 0.0000 | 0.0049 | 1.0000 | 0.2115 | 0.0448 | 2.0000 |
| **3:8** | 0.3029 | -0.0048 | -1.0000 | -0.0753 | 0.0057 | 1.3864 |
| **3:9** | 0.2726 | -0.0054 | -1.0000 | -0.0801 | 0.0064 | 1.3420 |
| **3:10** | 0.0000 | 0.0518 | 0.5294 | 0.5294 | 0.2802 | 2.0000 |
| **3:11** | 0.0000 | 0.0578 | 0.9098 | 0.7182 | 0.5158 | 2.0000 |
| **3:13** | 0.7057 | -0.0007 | -1.0000 | -0.0273 | 0.0007 | 1.7918 |
| **5:6** | 0.4675 | 0.0016 | 0.3671 | 0.0529 | 0.0028 | 2.0000 |
| **5:7** | 0.4675 | 0.0016 | 0.3671 | 0.0529 | 0.0028 | 2.0000 |
| **5:8** | 0.0000 | 0.0345 | 1.0000 | 0.4106 | 0.1686 | 2.0000 |
| **5:9** | 0.0000 | 0.0389 | 1.0000 | 0.4368 | 0.1908 | 2.0000 |
| **5:10** | 0.0000 | 0.0863 | 1.0000 | 0.6728 | 0.4527 | 2.0000 |
| **5:11** | 0.0000 | 0.0561 | 1.0000 | 0.5311 | 0.2821 | 2.0000 |
| **5:13** | 0.0000 | 0.0086 | 1.0000 | 0.2018 | 0.0407 | 2.0000 |
| **6:7** | 0.0000 | 0.0055 | 1.0000 | 1.0000 | 1.0000 | 2.0000 |
| **6:8** | 0.8281 | -0.0002 | -1.0000 | -0.0159 | 0.0003 | 1.9560 |
| **6:9** | 0.8173 | -0.0003 | -1.0000 | -0.0170 | 0.0003 | 1.9505 |
| **6:10** | 0.1877 | 0.0022 | 0.4414 | 0.0958 | 0.0092 | 2.0000 |
| **6:11** | 0.0715 | 0.0024 | 0.4645 | 0.1296 | 0.0168 | 2.0000 |
| **6:13** | 0.9150 | -0.0001 | -1.0000 | -0.0078 | 0.0001 | 1.9890 |
| **7:8** | 0.8281 | -0.0002 | -1.0000 | -0.0159 | 0.0003 | 1.9560 |
| **7:9** | 0.8173 | -0.0003 | -1.0000 | -0.0170 | 0.0003 | 1.9505 |
| **7:10** | 0.1877 | 0.0022 | 0.4414 | 0.0958 | 0.0092 | 2.0000 |
| **7:11** | 0.0715 | 0.0024 | 0.4645 | 0.1296 | 0.0168 | 2.0000 |
| **7:13** | 0.9150 | -0.0001 | -1.0000 | -0.0078 | 0.0001 | 1.9890 |
| **8:9** | 0.0000 | 0.0418 | 1.0000 | 0.9401 | 0.8838 | 2.0000 |
| **8:10** | 0.8877 | 0.0007 | 0.0170 | 0.0104 | 0.0001 | 1.7853 |
| **8:11** | 0.4167 | -0.0031 | -1.0000 | -0.0595 | 0.0035 | 1.5460 |
| **8:13** | 0.7580 | -0.0005 | -1.0000 | -0.0226 | 0.0005 | 1.9138 |
| **9:10** | 0.9903 | 0.0001 | 0.0014 | 0.0009 | 0.0000 | 1.7417 |
| **9:11** | 0.3875 | -0.0035 | -1.0000 | -0.0633 | 0.0040 | 1.5053 |
| **9:13** | 0.7431 | -0.0005 | -1.0000 | -0.0240 | 0.0006 | 1.9033 |
| **10:11** | 0.0000 | 0.0636 | 1.0000 | 0.7894 | 0.6231 | 2.0000 |
| **10:13** | 0.0000 | 0.0098 | 1.0000 | 0.3000 | 0.0900 | 2.0000 |
| **11:13** | 0.0000 | 0.0102 | 1.0000 | 0.3801 | 0.1444 | 2.0000 |
